# Goats without Prion Protein Display Enhanced Proinflammatory Pulmonary Signaling and Extracellular Matrix Remodeling upon Systemic Lipopolysaccharide Challenge

**DOI:** 10.3389/fimmu.2017.01722

**Published:** 2017-12-06

**Authors:** Øyvind Salvesen, Malin R. Reiten, Jorke H. Kamstra, Maren K. Bakkebø, Arild Espenes, Michael A. Tranulis, Cecilie Ersdal

**Affiliations:** ^1^Faculty of Veterinary Medicine, Department of Production Animal Clinical Sciences, Norwegian University of Life Sciences, Sandnes, Norway; ^2^Faculty of Veterinary Medicine, Department of Basic Sciences and Aquatic Medicine, Norwegian University of Life Sciences, Oslo, Norway

**Keywords:** acute lung injury, cellular prion protein, systemic inflammation, lipopolysaccharide, innate immunity, RNA sequencing, extracellular matrix remodeling, histopathology

## Abstract

A naturally occurring mutation in the *PRNP* gene of Norwegian dairy goats terminates synthesis of the cellular prion protein (PrP^C^), rendering homozygous goats (*PRNP*^Ter/Ter^) devoid of the protein. Although PrP^C^ has been extensively studied, particularly in the central nervous system, the biological role of PrP^C^ remains incompletely understood. Here, we examined whether loss of PrP^C^ affects the initial stage of lipopolysaccharide (LPS)-induced acute lung injury (ALI). Acute pulmonary inflammation was induced by intravenous injection of LPS (*Escherichia coli* O26:B6) in 16 goats (8 *PRNP*^Ter/Ter^ and 8 *PRNP*^+/+^). A control group of 10 goats (5 *PRNP*^Ter/Ter^ and 5 *PRNP*^+/+^) received sterile saline. Systemic LPS challenge induced sepsis-like clinical signs including tachypnea and respiratory distress. Microscopic examination of lungs revealed multifocal areas with alveolar hemorrhages, edema, neutrophil infiltration, and higher numbers of alveolar macrophages, with no significant differences between *PRNP* genotypes. A total of 432 (*PRNP*^+/+^) and 596 (*PRNP*^Ter/Ter^) genes were differentially expressed compared with the saline control of the matching genotype. When assigned to gene ontology categories, biological processes involved in remodeling of the extracellular matrix (ECM), were exclusively enriched in PrP^C^-deficient goats. These genes included a range of collagen-encoding genes, and proteases such as matrix metalloproteinases (*MMP1, MMP2, MMP14, ADAM15*) and cathepsins. Several proinflammatory upstream regulators (TNF-α, interleukin-1β, IFN-γ) showed increased activation scores in goats devoid of PrP^C^. In conclusion, LPS challenge induced marked alterations in the lung tissue transcriptome that corresponded with histopathological and clinical findings in both genotypes. The increased activation of upstream inflammatory regulators and enrichment of ECM components could reflect increased inflammation in the absence of PrP^C^. Further studies are required to elucidate whether these alterations may affect the later reparative phase of ALI.

## Introduction

The cellular prion protein (PrP^C^) is an evolutionarily conserved protein abundantly expressed in the central nervous system, and also at moderate levels peripherally, such as in the immune system and lungs (1–4). Misfolding of PrP^C^ is the essential step for the development of prion disorders in humans and animals (5). Although intensively studied for several decades, the normal functions of PrP^C^ have proven difficult to identify. Not surprisingly, the main focus has been on the nervous system, and PrP^C^ has been linked to neuroprotection, signal transduction, and myelin maintenance, among others (6, 7). More recently, the association of PrP^C^ with inflammatory responses and the immune system has been investigated. Lack of PrP^C^ enhances the inflammatory reaction in several models and organ systems (8–11), and it has been proposed that PrP^C^ plays important roles in neuroimmune cross-talk (12, 13).

In a line of Norwegian dairy goats, a mutation in the PrP^C^-encoding gene (*PRNP*) terminates PrP^C^ synthesis. Animals homozygous for this mutation (*PRNP*^Ter/Ter^) are the only known non–transgenic mammals devoid of PrP^C^ (14). The goats are postulated to be scrapie-resistant, and constitute a unique animal model for studying PrP^C^ physiology. No major loss-of-function phenotypes have been observed under normal herd conditions, but a hematological shift in red blood cells (15) and a primed state of type I interferon-stimulated genes are present at rest in these animals (16). When exposed to systemic lipopolysaccharide (LPS), PrP^C^-deficient goats display a prolonged sickness behavior and a slightly different immunological profile that is skewed toward a type I interferon response (17).

Acute lung injury (ALI) and its most severe manifestation, acute respiratory distress syndrome, contribute to increased morbidity and mortality in critically ill patients (18, 19). The most common cause of ALI is sepsis caused by a systemic bacterial infection (20). Early events in this syndrome can be mimicked by administration of LPS, a component of the outer membrane of Gram-negative bacteria. LPS is recognized by the toll-like receptor 4 (TLR4) complex expressed by innate immune cells and also by other cells such as endothelial cells within the lung (21). Systemic administration of LPS initially induces apoptosis of the alveolar capillary endothelium (22) and epithelium (23), and neutrophils are rapidly recruited. The migration of neutrophils into the alveoli is dependent upon crossing the basement membrane, possibly through gaps in the basal laminae (24), and is facilitated by proteolytic degradation of the highly organized extracellular matrix (ECM) (25). Once at the site of injury, activated macrophages and infiltrating neutrophils release a variety of proinflammatory cytokines, enzymes, and reactive oxygen and nitrogen species. These molecules participate in maintaining the inflammatory state and defeating invading pathogens, but also inflict damage of the lung parenchyma and microvascular structure. This results in proteinaceous edema and hemorrhage, as seen in the exudative phase of ALI (26). To the best of our knowledge, no studies have used RNA sequencing to investigate the pulmonary inflammatory response in mammals upon systemic LPS challenge.

We hypothesized that PrP^C^-deficient goats are more susceptible to inflammatory stimuli, and therefore examined the responsiveness to LPS of *PRNP*^Ter/Ter^ goats, compared with normal goats (*PRNP*^+/+^). This study shows a full-scale transcriptional profile coupled to morphological alterations in caprine lungs, an organ highly susceptible to systemic inflammation and investigates whether loss of PrP^C^ affects the initial stage of ALI.

## Materials and Methods

### Animals

A total of 26 Norwegian dairy goat kids, 13 *PRNP*^Ter/Ter^ and 13 *PRNP*^+/+^ animals, were included in the study. Details about housing conditions can be found in reference (17) and an overview of the study groups including treatment, animal number, age, weight, and gender can be found in Table S1 in Supplementary Material. The animal experiment was performed in compliance with ethical guidelines and approved by the Norwegian Animal Research Authority (ID 5827, 6903, and 7881) with reference to the Norwegian regulation on animal experimentation (FOR-2015-06-18-761).

### Experimental Protocol

Sixteen goats (8 *PRNP*^Ter/Ter^ and 8 *PRNP*^+/+^) received a dual dose of LPS (*Escherichia coli* O26:B6, L2654 Sigma-Aldrich, USA) intravenously with a 24-h time interval between doses; 0.1 µg/kg (day 1) and 0.05 µg/kg (day 2). The control group consisted of 10 goats (5 *PRNP*^Ter/Ter^ and 5 *PRNP*^+/+^) and were given corresponding volumes of sterile saline. Clinical examinations, including respiratory rate, were performed at 12 time points during the first 7 h of day 1 and at 9 time points after the second LPS injection. Additionally, episodes of coughing and panting were recorded. The animals were euthanized by an overdose of pentobarbital 5 h after the second LPS challenge.

### Gross Pathology and Histopathology Score

#### Gross Pathology

After euthanasia, goats were fully necropsied. The amount of fluid in the thoracic cavity and airways, and the distribution and amount of hemorrhages, atelectasis, edema, and emphysema in the lungs were recorded. Additionally, macroscopic changes compatible with lungworm (*Muellerius capillaris*) were noted.

#### Histopathology

Tissues were collected at standardized areas dorsally and ventrally from the left caudal lung lobe, as well as from areas with gross pathology, mainly hemorrhages. Tissues were immersion fixed in 4% formaldehyde for 1 week and then dehydrated in graded ethanol and paraffin embedded. Paraffin sections (4 µm) were mounted on slides and stained with hematoxylin and eosin (HE). Martius-scarlet-blue (MSB) staining was performed on selected sections to evaluate fibrin deposition. The HE sections were examined by light microscopy and a blinded, semiquantitative evaluation was performed to assess lung pathology. Both the airways and the lung interstitium were evaluated, focusing on the alveolar lumen and septa. Evaluation parameters included hemorrhage, hyperemia, fibrin deposition, necrosis, and neutrophil and macrophage infiltration. Pathological changes were scored as follows: 0 = minimal; 1 = little/a few, 2 = moderate; 3 = severe, including half-step grading. A total lung histopathology score was calculated for each section by summarizing the values from the above mentioned criteria.

### Immunohistochemistry (IHC)

#### 6H4 and SAF32

Frozen lung tissue from a control *PRNP*^+/+^ goat was used to evaluate the pulmonary distribution of PrP^C^. After dissection, lung tissues were frozen in isopentane, transferred to liquid nitrogen, and stored at −70°C. Sections (8 µm) were cut on a cryostat and mounted on Superfrost^®^ Plus slides (Menzel-Gläser, Thermo Scientific). The sections were fixed in formol-calcium, followed by 5 min inhibition of endogenous peroxidase activity (DAKO Peroxidase Block, Agilent). After blocking in normal goat serum (1:50) for 10 min, the sections were incubated with the primary anti-PrP antibodies 6H4 (1:500, Prionics AG) or SAF32 (1:1,000, Cayman) for 1 h at room temperature. Further steps were performed with the Envision + System-HRP DAB (Dako, K4007), according to the manufacturer’s instruction. Antibody specificity was checked by performing the same procedure on lung sections from a *PRNP*^Ter/Ter^ goat.

#### S100A8

Formalin-fixed, paraffin-embedded sections (4 µm) were mounted on Superfrost^®^ Plus slides (Menzel-Gläser, Thermo Scientific) and dried for 30 min at 58°C. The sections were deparaffinized in xylene and rehydrated through decreasing concentrations of graded ethanol. The PT link system (100°C for 15 min in Tris/EDTA-buffer, pH 9.1) was used for epitope retrieval. Endogenous peroxidase activity was blocked by incubation in 3% H_2_O_2_ in methanol for 20 min at room temperature. Sections were then blocked in normal goat serum (1:50), diluted in phosphate-buffered saline (PBS) and incubated with the primary antibody S100A8 (1:3,200, Abcam) for 1.5 h at room temperature. Further steps were performed with EnVison + System-HRP AEC (Dako, K4009).

Washing between steps was performed with PBS for both procedures. Sections were counterstained in hematoxylin, and mounted using Aquatex medium (Merck). All runs included a negative control section in which the primary antibody was replaced with 1% PBS. The number of cells that were positive for the S100 calcium-binding protein A8 (S100A8) was quantified within the field of view of three representative areas in each animal/section (400× magnification, ø = 450 µm).

### RNA Extraction, Quality Control, and Pooling

Tissue samples were collected at a standardized area dorsally from the left caudal lung lobe within 15 min after euthanasia, and immediately immersed in RNA later (Invitrogen) and stored at −70°C. RNA was extracted from approximately 30 mg of lung tissue using the RNeasy Lipid Tissue Mini Kit (Qiagen, 74804) according to the manufacturer’s instruction. High quality RNA (RNA integrity number > 8) from individuals was pooled in equal amounts per treatment and genotype, reaching a final amount of 15 μg, as described in Salvesen et al. (17). RNA quality data are summarized in Table S2 in Supplementary Material.

### RNA Sequencing and Differential Expression Analyses

RNA samples were shipped on dry ice to Novogene (Hong Kong) for RNA sequencing. Paired-end 150 bp sequencing was performed on an Illumina HiSeq2000. Quality control summary and mapping status are given in Tables S3 and S4 in Supplementary Material. Normalization was performed by the trimmed mean of M values method, *p*-value estimation assuming Poisson distribution, and false discovery rate (FDR) estimation by Benjamini-Hochberg correction of multiple testing (27, 28). Differential gene expression analysis was performed using DEGSeq (28) with the following criteria: Log2 ratio ± 0.59 (fold change ± 1.5) and a FDR-adjusted *q*-value (*q* < 0.05). The genomic response to LPS in each *PRNP* genotype was assessed by comparing LPS-groups with the saline-treated control of the matching genotype. Fragments per kilobase of exon per million fragments mapped values, which take into account the effects of both sequencing depth and gene length, were used to estimate gene expression levels.

### Ingenuity Pathway Analysis (IPA) and Gene Ontology (GO) Enrichment

Gene lists from *Capra hircus* were first converted to gene symbols using the NCBI batch converter tool.^1^ Pathway analysis was performed with both IPA (v. 36601845) and GO analysis with the online DAVID 6.8 software (29, 30). Because the *C. hircus* genome is not available in both analyses, *Homo sapiens* orthologs were used instead. The lists of differentially expressed genes (DEGs), containing gene identifiers and corresponding expression values, were uploaded into the IPA software (Qiagen). The top enriched canonical pathways, upstream regulators and gene networks were identified by the “core analysis” function, using a cut-off in absolute fold change of 1.5 and a FDR < 0.05. The IPA software uses the Fishers exact test for pathway enrichment and *Z*-scores for pathway activation. A Benjamini-Hochberg adjusted *p*-value < 0.05 was considered significant for GO analysis. DEGs of the two genotypes were analyzed separately.

### Validation of RNA-seq by Real-time qPCR

Complementary DNA was generated using RNA from each individual sample by the QuantiTect Reverse Transcription Kit (Qiagen) according to manufacturer’s instructions. A non-reverse transcriptase control and no template control were included. The expression of nine representative target genes [serum amyloid A3.2 (*SAA3.2*), *CXCL10, ITGAM, CD14, IFI6, GADPH, S100A9, PRNP*, and interleukin 1B (*IL1B*)] was investigated by the Light cycler 480 qPCR system. Real-time reactions were prepared using SYBR Green PCR Master Mix including 10 µl cDNA (1:10) in each reaction, and qPCR was performed with standard cycling conditions: initial denaturation for 5 min at 95°C, followed by 40 amplification cycles (10 s at 95°C, 10 s at 60°C, and 15 s at 72°C) and construction of melting curves. A standard curve was generated for each target gene to obtain primer amplification efficiencies, correlations, and dynamic range. Normalization was performed against the *ACTB* reference gene, which showed no significant change by LPS-treatment in the RNA-seq data, confirming stability as internal control. Relative expression was calculated using the 2^−ΔΔCq^ method as described in Ref. (31). Primer sequences are given in Table S5 in Supplementary Material.

### Descriptive and Statistical Analyses

Data are presented as mean ± SEM. Graphical and statistical analyses were performed in GraphPad Prism 6 (GraphPad Software Inc., USA) and Microsoft Excel 2013. A one-way ANOVA, followed by Dunnetts’s *post hoc* test, was performed when comparing three or more groups. Differences in lung histopathology score between two groups were assessed by the Mann–Whitney *U*-test. Significance levels *p* < 0.05; *p* < 0.01; *p* < 0.001 are indicated.

## Results

### Systemic LPS Challenge Induces Tachypnea and Respiratory Distress

**Figure 1:**
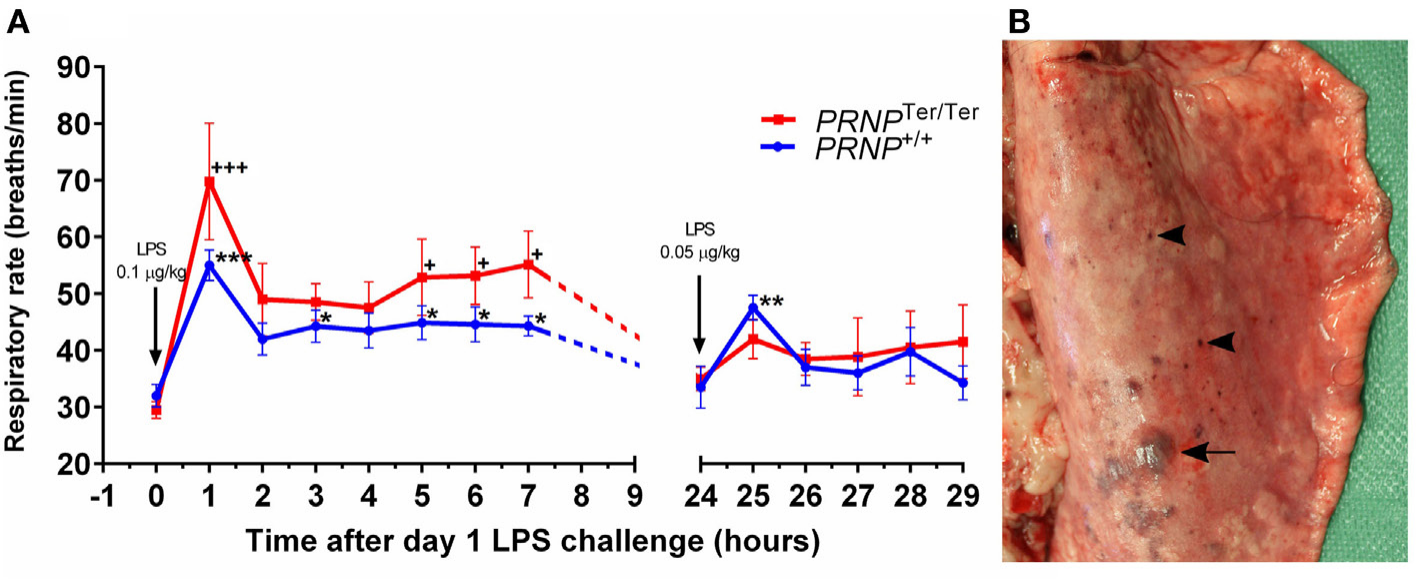
Respiratory rate and lung gross pathology after systemic lipopolysaccharide (LPS) challenge. Goats were subjected to a dual LPS challenge; 0.1 and 0.05 µg/kg with a 24-h time interval between doses. **(A)** The diagram shows the respiratory rate (breaths/min) after LPS challenge. Values are mean ± SEM. Symbols * and + represent significantly increased respiratory rate compared with baseline samples (0 h) of the matching genotype. **(B)** Dorsal view of the right caudal lung lobe. Subplural ecchymosis (arrow) and petechial hemorrhages (arrowhead) display a multifocal distribution.

After LPS challenge, all eight goats from both genotypes displayed sepsis-like clinical signs, such as sickness behavior, fever, and tachycardia. PrP^C^-deficient goats displayed prolonged sickness behavior, and hematological analysis showed a rapid and profound leukopenia in both genotypes as we have previously described (17). Respiratory rate increased significantly in both *PRNP* genotypes after the initial high dose of LPS (0.1 µg/kg), peaking 1 h post-LPS challenge, and remained elevated at all subsequent time points on day 1 (Figure 1A). The mean respiratory rate was higher in the *PRNP*^Ter/Ter^ group at all time points during the first day, albeit not statistically significant. Periods of heavy abdominal breathing, coughing, and panting were observed in seven out of eight animals of both genotypes receiving LPS, whereas none of the control animals displayed alterations in clinical parameters. Clinical signs of respiratory distress were less profound on day 2, when the dosage of LPS was halved.

### Systemic LPS Challenge Induces Circulatory Disturbances and Inflammation

#### Gross Pathology

Multifocal ecchymosis and petechial hemorrhages were observed in four of eight *PRNP*^+/+^ goats and five of eight *PRNP*^Ter/Ter^ goats receiving LPS. The majority of hemorrhages were subpleural and dorsally located in the caudal lung lobes (Figure 1B). Two *PRNP*^Ter/Ter^ animals had hydrothorax with 90 and 400 mL of serous fluid within the thoracic cavity. Both these goats had mild to moderate ventral atelectasis. Four animals (*PRNP*^Ter/Ter^) had sparse to moderate amounts of froth and fluid within the trachea and main bronchi, and one animal had a parenchymal lung edema, characterized by a wet cut surface. Sparse lungworm-changes (*M. capillaris*) were equally distributed between the genotypes in both the LPS and control groups.

#### Microscopic Changes

**Figure 2:**
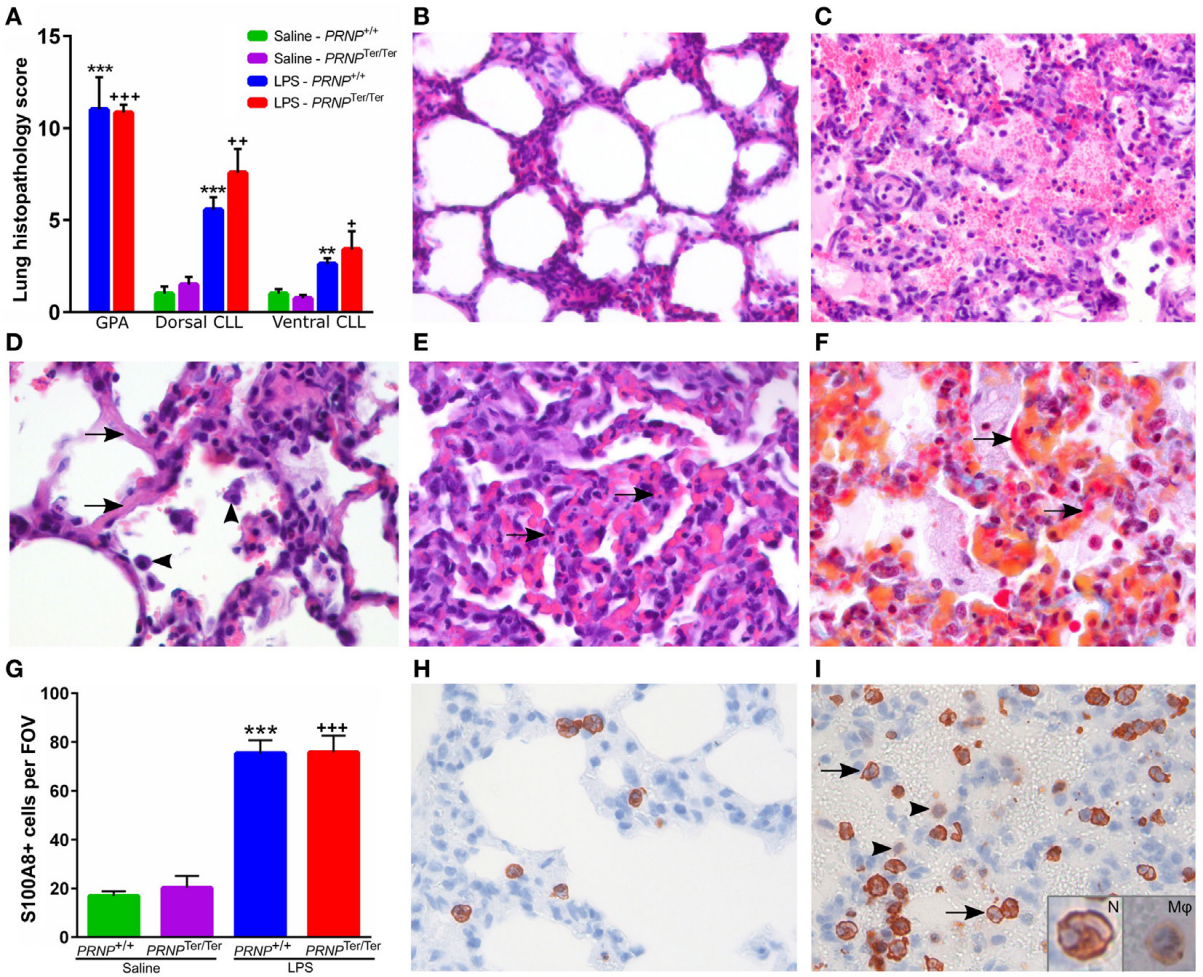
Histopathology after lipopolysaccharide (LPS) challenge. **(A)** A blinded scoring of lung histopathology was performed as described in methods. Sections from the dorsal caudal lung lobe (CLL) and ventral CLL were scored in all animals, in addition to sections from areas with gross pathology, mainly hemorrhages (GPA). Symbols represent significantly increased lung histopathology score compared with saline controls of the matching genotype. GPA is compared with the dorsal CLL of the matching control. **(B)** Normal lung tissue from a saline control goat. **(C)** Alveolar edema and hemorrhages accompanied by neutrophil infiltration and increased number of alveolar macrophages. **(D)** Hyaline septa (arrows) and increased number of alveolar macrophages (arrowheads). **(E)** Area with moderate atelectasis and hyperemia. Several single cell necroses (arrows) can be seen within the alveolar septa. **(F)** MSB staining shows deposition of fibrin within the septa (arrows) and small amounts in the alveoli. Sections **(C–F)** are from areas with pathology in LPS-treated animals. **(G)** Mean number of S100A8-positive cells quantified within a representative 400× field of view (FOV, ø = 450 µm). Symbols represent significantly increased levels compared with saline controls of the matching genotype. **(H)** Normal lung tissue from a control animal displaying a few S100A8-positive neutrophils within the alveolar septa. **(I)** After LPS challenge, increased numbers of S100A8-positive neutrophils are sequestered within the septal capillaries and some infiltrate the alveoli (arrows). Alveolar macrophages displayed a weak S100A8-signal (arrowheads). Insertions show a magnified neutrophil (N) with segmented nucleus and an alveolar macrophage (Mφ). Magnification: **(B,C)** 200×; **(D–I)** 400×. Values are mean ± SEM.

The lung histopathology scores (Figure 2A) were significantly higher in the LPS-groups than in the saline controls (Figure 2B). Multifocal microscopic hemorrhages were recorded in seven out of eight animals receiving LPS in both genotypes, located both subpleurally and deeper within the lung parenchyma. These areas were characterized by alveolar hemorrhage and edema, increased amount of alveolar macrophages, and infiltration of neutrophils (Figure 2C). Some hyaline membranes were observed lining the alveolar wall (Figure 2D). Focally, an increased number of neutrophils were present in the alveolar septa, accompanied by single cell necrosis and hyperemia (Figure 2E). In these same foci, MSB staining showed fibrin deposition along alveolar septa of a few LPS-treated animals (Figure 2F). Immunohistochemical labeling of S100 calcium binding protein A8 (S100A8) increased upon LPS challenge, and was predominantly expressed by neutrophils, and at lower levels, in alveolar macrophages (Figures 2G–I). The histopathological changes were more profound dorsally than ventrally. There were no significant differences between *PRNP* genotypes.

### *PRNP* Expression and Cellular Distribution of PrP^C^

**Figure 3:**
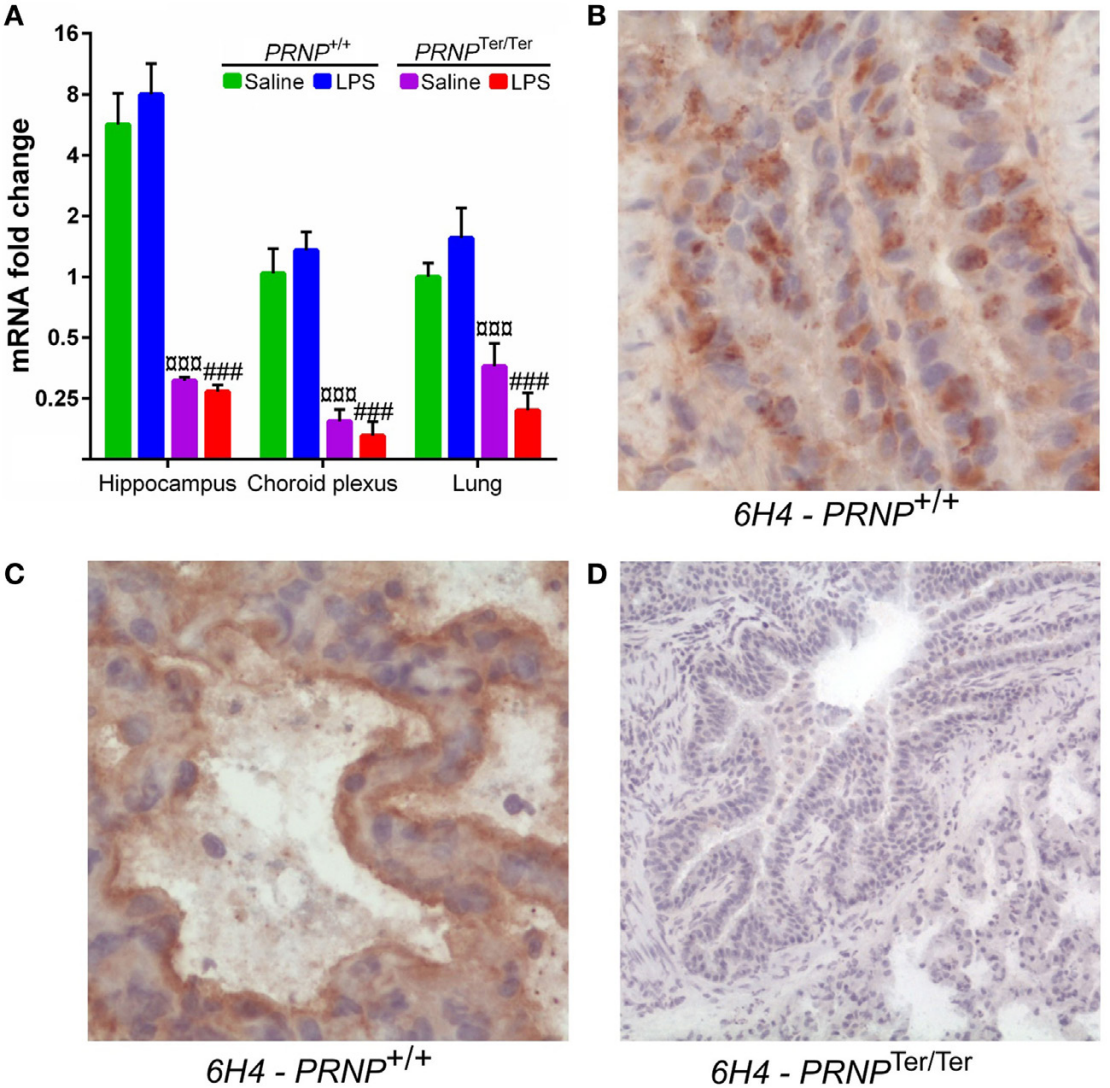
*PRNP* expression and cellular distribution of PrP^C^. **(A)** Comparison of *PRNP* expression in three tissues from Norwegian dairy goats before and after lipopolysaccharide challenge. Symbols represent significantly (*p* < 0.001) reduced expression in *PRNP*^Ter/Ter^ goats, compared with *PRNP*^+/+^ goats of the same treatment. **(B)** PrP^C^ shows a granular distribution within bronchial epithelial cells. **(C)** In the alveoli, PrP^C^ labeling is associated with the luminal surface of the alveolar septa, probably associated with alveolar pneumocytes. **(D)** No PrP^C^ was detected in sections from *PRNP*^Ter/Ter^ goats. All sections are from representative areas stained with the 6H4 antibody. Magnification **(B,C)** 400×; **(D)** 100×.

The level of *PRNP* expression in the lungs was similar to that of the choroid plexus, and five times lower than in the hippocampus (17). After LPS challenge, *PRNP* expression increased by 1.6-fold in the lungs of *PRNP*^+/+^ goats, albeit not significantly. Expression of *PRNP* is reduced to about 25% in *PRNP*^Ter/Ter^ goats (Figure 3A). The localization of PrP^C^ in the lungs of non-treated animals was investigated by use of 6H4 and SAF32 antibodies. A granular distribution of PrP^C^ was observed within bronchial and bronchiolar epithelial cells of *PRNP*^+/+^ goats (Figure 3B). In the alveoli, PrP^C^ was primarily localized at the luminal side of the alveolar septa, probably associated with alveolar pneumocytes (Figure 3C). No PrP^C^ was detected in *PRNP*^Ter/Ter^ goats, confirming the absence of synthesized protein as well as the specificity of the antibodies (Figure 3D). Staining with SAF32 showed similar results (Figure S1 in Supplementary material).

### The Lung Transcriptome Is Profoundly Altered by Systemic LPS Challenge

**Figure 4:**
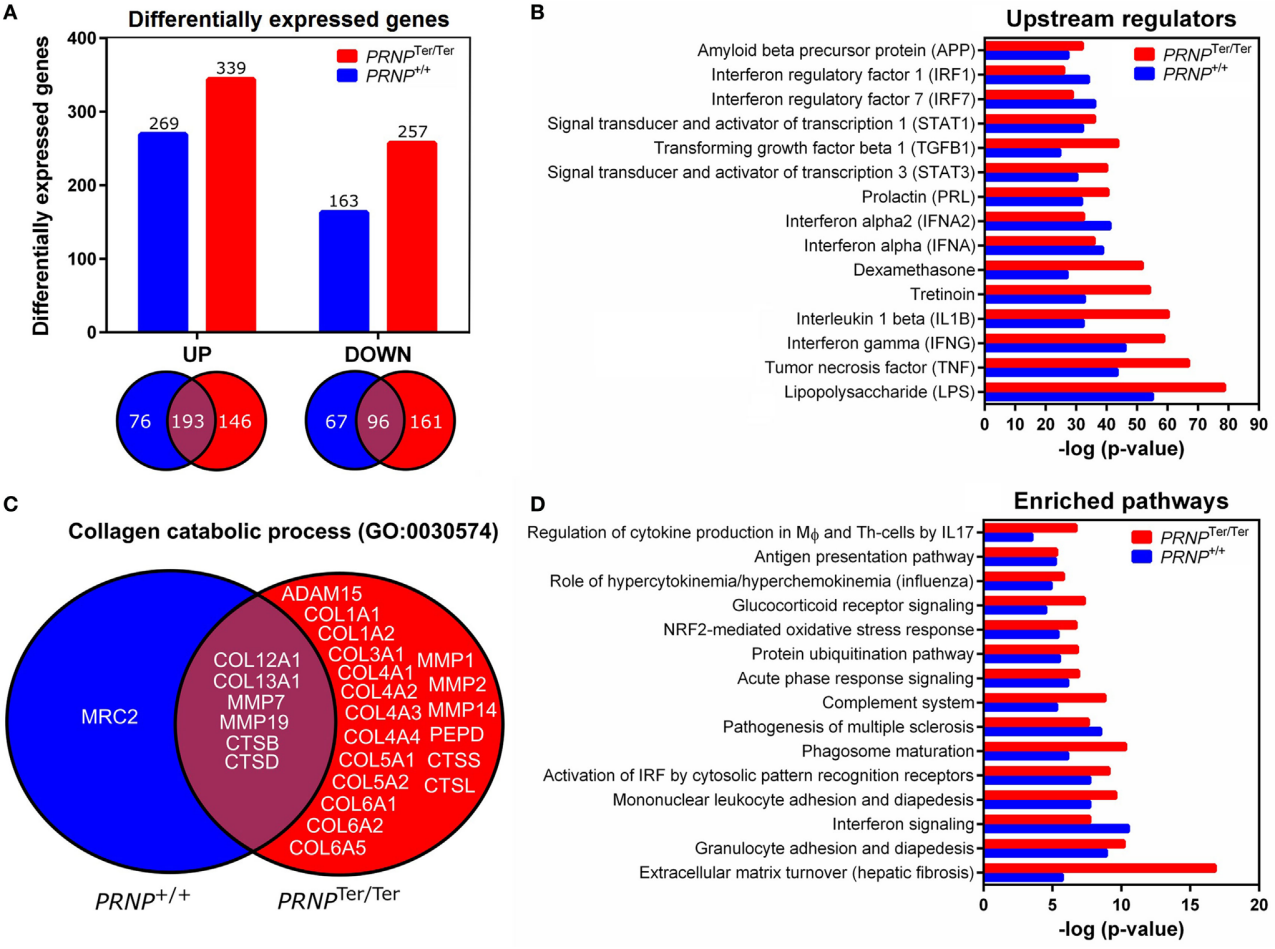
Differentially expressed genes after LPS challenge and biological function analysis. **(A)** Differential expression analyses were performed by comparing the LPS-groups to the saline control group of the matching genotype (log2 ratio 0.59 and *q*-value < 0.05). Venn diagrams show genes that overlap between the two genotypes. **(B)** Shown are the top 15 overrepresented upstream regulators identified by ingenuity pathway analysis (IPA). **(C)** Venn diagram showing the distribution of genes characterized as collagen catabolic process (GO:0030574) in the two *PRNP* genotypes. **(D)** The top 15 affected pathways identified by IPA are shown.

After LPS challenge, a total of 432 [upregulated: 269, downregulated: 163] and 596 (upregulated: 339, downregulated: 257) genes were differentially expressed compared to the saline control group in normal and PrP^C^-deficient goats, respectively (Data Sheet S1 in Supplementary Material). The Venn diagrams show that 146 upregulated and 161 downregulated genes exclusively belonged to the PrP^C^-deficient goats when the filtration criteria (log2 ratio 0.59, *q* < 0.05) were applied (Figure 4A). However, closer investigation of the transcriptional data revealed that 95% of the DEGs were regulated in the same direction in both genotypes, albeit some excluded by fold change or *q*-value. Genes upregulated by a log2 ratio ≥ 3 (fold change ≥ 8) after LPS challenge are listed in Table 1. The most upregulated gene in both *PRNP* genotypes was the acute phase protein *SAA3* (51-fold, *PRNP*^Ter/Ter^; 25-fold, *PRNP*^+/+^). Other highly upregulated genes were cytokines, chemokines, and metal-binding proteins such as metallothioneins and several S100 proteins. The most downregulated gene was *CYP1A1* (24-fold, *PRNP*^Ter/Ter^; 22-fold, *PRNP*^+/+^), a member of the cytochrome P450 superfamily (Data Sheet S1 in Supplementary Material).

**Table 1:** Genes upregulated by log2 ratio ≥ 3 (fold change ≥ 8) after LPS challenge.

Genes upregulated by a log2 ratio ≥ 3 in both genotypes			Log2 ratio (LPS vs. saline)	

Gene ID	Symbol	Gene name	*PRNP*^**+**/**+**^	*PRNP*^Ter/Ter^
100860781	SAA3	Serum amyloid A3	4.66	5.69
102188618	MT1A	Metallothionein-1A	4.64	4.80
102174322	FOLR1	Folate receptor alpha	4.27	4.56
102188072	MT2	Metallothionein-2	4.14	3.75
102168428	SAA3.2	Serum amyloid A3.2 protein	3.90	4.63
102177100	ISG20	Interferon-stimulated exonuclease gene 20	3.73	3.09
102182273	S100A9	S100 calcium-binding protein A9	3.22	4.19
**Genes upregulated (log2 ratio ≥ 3) in ***PRNP***^Ter/Ter^ goats**				
102168876	S100A8	S100 calcium-binding protein A8	2.76	3.67
102173634	GPNMB	Glycoprotein nmb	ND	3.55
102175845	S100G	S100 calcium-binding protein G	ND	3.44
102169149	S100A12	S100 calcium-binding protein A12	2.27	3.17
**Genes upregulated (log2 ratio ≥ 3) in ***PRNP***^+/+^ goats**				
102169982	ISG15	ISG15 ubiquitin-like modifier	3.53	2.39
102189279	ASIC1	Acid sensing ion channel subunit 1	3.23	2.25
102190797	RSAD2	Radical S-adenosyl methionine domain containing 2	3.19	2.68
102187309	CXCL11	C-X-C motif chemokine ligand 11	3.16	2.34
102183403	CLEC4E	C-type lectin domain family 4 member E	3.06	2.97

*Differential expression analyses were performed by comparing LPS-treated groups to the saline control group of the matching genotype*.

*ND, not differentially expressed*.

### Affected Pathways Are Linked to LPS Challenge and Indicate Enhanced Inflammation and Turnover of ECM in PrP^C^-Deficient Goats

To assess the function of the DEGs, enriched pathways, networks and upstream regulators were identified by IPA. DEGs were also assigned to GO classifications, including biological process, cellular compartment and molecular function. In general, there was a high degree of overlap in the transcriptional response to LPS in normal and PrP^C^-deficient goats. As expected, a considerable amount of the DEGs belonged to the innate immune response and inflammatory response (Data Sheet S2 in Supplementary Material), and LPS was identified as the most significant upstream regulator of both genotypes (Figure 4B). The associated *p*-values indicate more enrichment of genes involved in the LPS-response in *PRNP*^Ter/Ter^ goats, particularly those stimulated by the proinflammatory cytokines IL-1β, IFN-γ, and TNF-α (Figure 4B). Other notably affected pathways in both genotypes involved acute-phase response signaling, antigen presentation, and granulocyte adhesion and diapedesis (Figure 4D). Although some upstream regulators of interferons were more significantly enriched in PrP^C^-deficient goats, predicted activation of interferon signaling were lower than in the normal goats. The full IPA output can be found in Data Sheet S3 in Supplementary Material.

**Figure 5:**
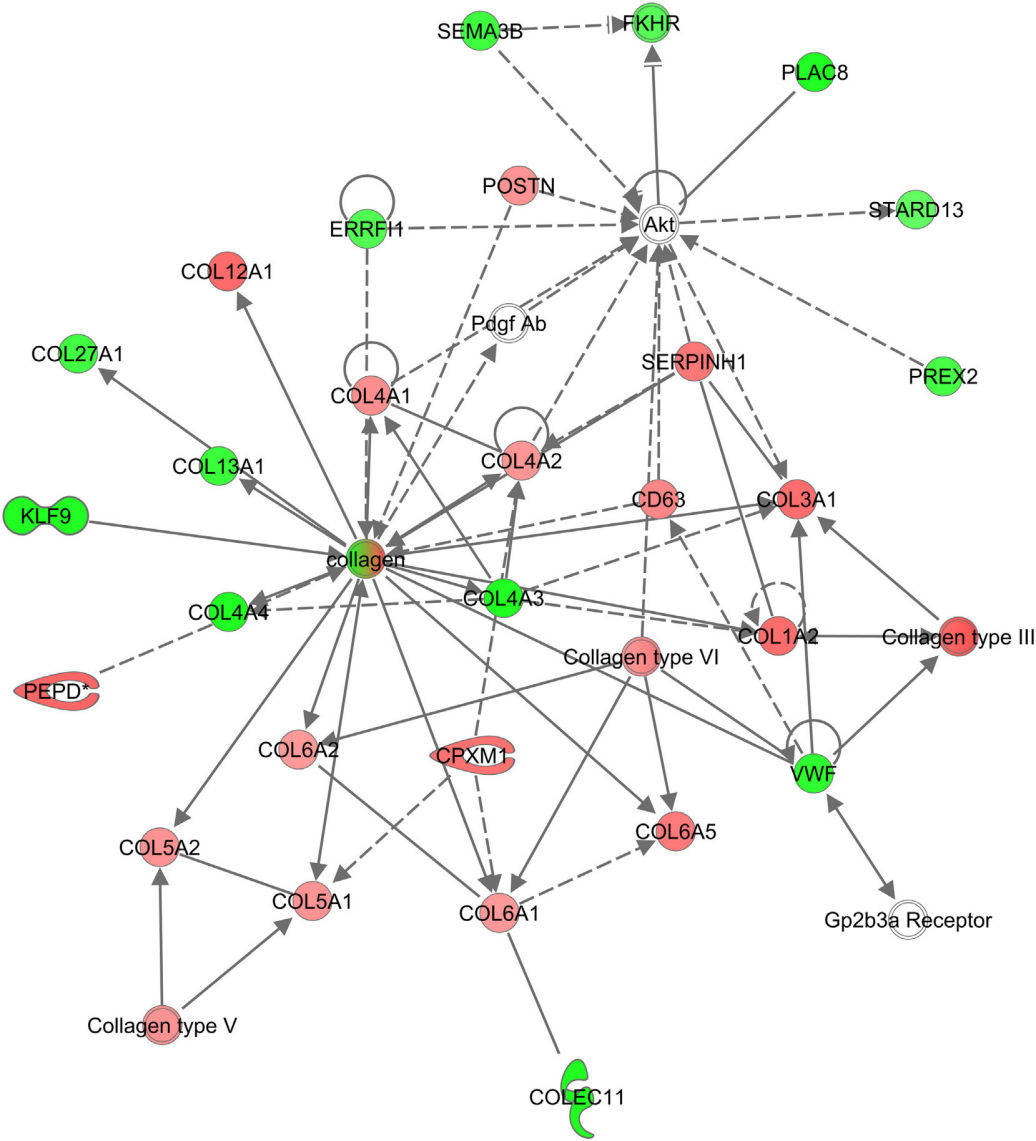
Network of genes related to connective tissue disorders in *PRNP*^Ter/Ter^ goats. An overrepresented network is shown exclusively for *PRNP*^Ter/Ter^ goats of differentially expressed genes and interacting partners related to connective tissue disorders. Red means upregulated, green means downregulated.

Several of the GO-terms, such as collagen catabolic process (GO:0030574), ECM disassembly (GO0022617), and ECM structural constituents (GO:0005201) were exclusively enriched in PrP^C^-deficient goats (Data Sheet S2 in Supplementary Material). The genes were primarily in an upregulated state, and included collagen-encoding genes, secreted metalloproteinases, such as matrix metalloproteinases (*MMPs*) and *ADAM* genes, as well as cathepsins primarily located within lysosomes (Figure 4C). In line with this, the top canonical pathway in *PRNP*^Ter/Ter^ goats was related to turnover of ECM (Figure 4D). Also, IPA analysis revealed a significantly enriched network involved in connective tissue disorders (−log*P* 39, Figure 5).

### Validation of RNA-seq Data by qPCR

**Figure 6:**
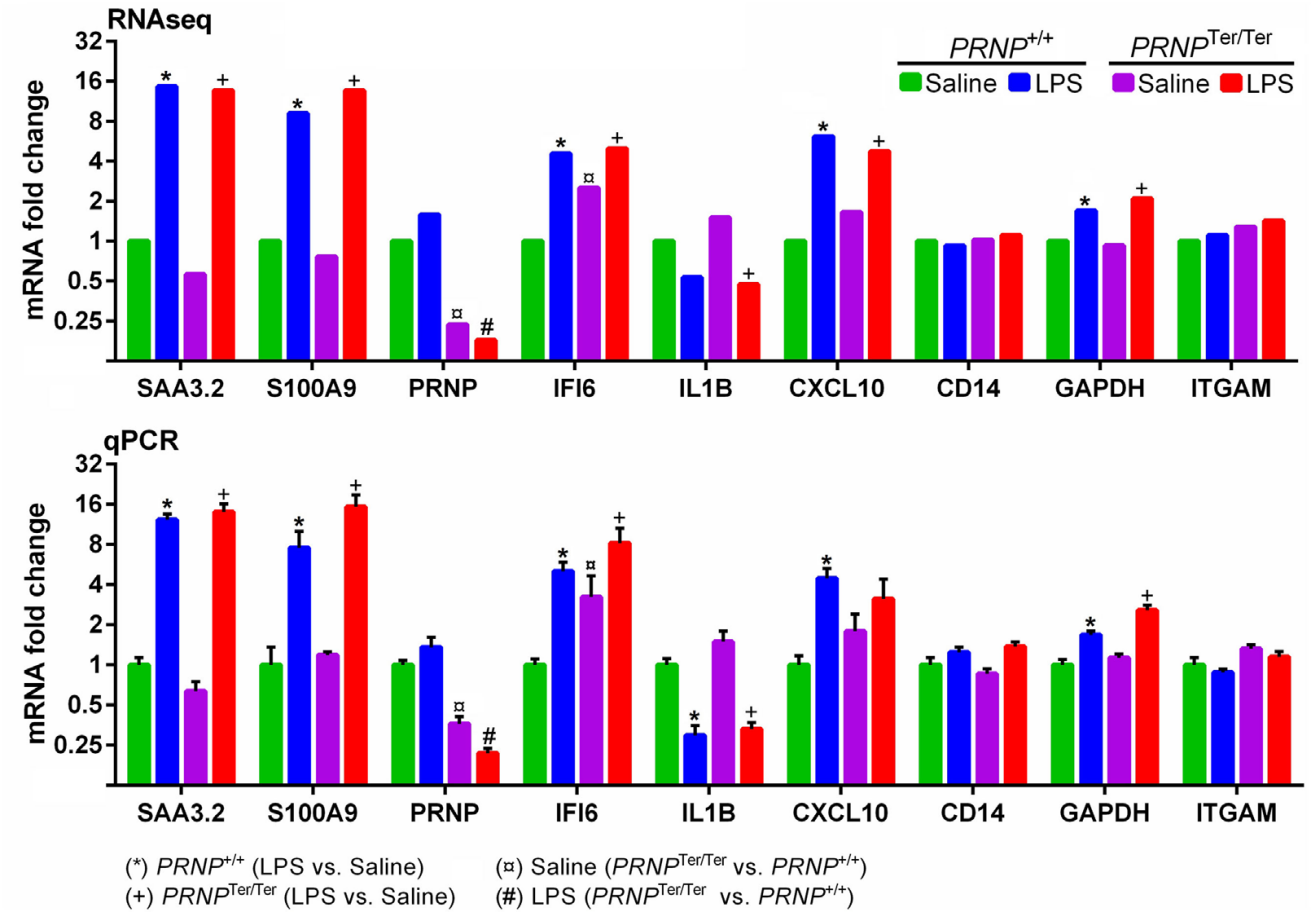
Validation of RNA-seq data by qPCR. Nine target genes representing both differentially expressed and non-differentially expressed genes were measured by qPCR. For each gene, the expression levels are normalized relative to the saline-treated *PRNP*^+/+^ group. RNA-seq symbols above bars represent a significant differential expression (log2 ratio ± 0.59, *q* < 0.05). Bars (qPCR) display mean expression ± SEM with symbols representing significant differences, as assessed by multiple *t*-tests (*p* < 0.05).

The expression of nine target genes (*SAA3.2, CXCL10, ITGAM, CD14, IFI6, GADPH, S100A9, PRNP*, and *IL1B*) was investigated by qPCR on individual RNA samples from lung tissue (Figure 6). A strong correlation (*r* = 0.975, *p* < 0.0001, Pearson correlation) was observed between log2-transformed expression values of RNA-seq and qPCR, in line with our previous reports from other tissues (16, 17). Differential expression was confirmed by qPCR in 34 out of 36 comparisons.

## Discussion

We performed an LPS challenge in goats with and without PrP^C^ and characterized the clinical, morphological, and transcriptional response to acute pulmonary inflammation. Both genotypes displayed tachypnea, clinical signs of respiratory distress, and histopathological evidence of ALI. A significant enrichment of ECM turnover, along with an increased activation score of genes related to the proinflammatory LPS response was observed in PrP^C^-deficient goats.

In the current study, more than 70% of the DEGs classified as being involved in collagen catabolic processes belonged exclusively to *PRNP*^Ter/Ter^ goats, and the connective tissue disorder network was significantly enriched. The collagen-encoding genes were primarily upregulated, probably as a result of resident fibroblast activation mediated by transforming growth factor (TGF)-β (32, 33). In line with this, the upstream regulator TGF-β showed an increased activation score in PrP^C^-deficient goats. Indeed, PrP^C^ has been reported to modulate B1 integrin signaling directly (34, 35), which is a key regulator of TGF-β-dependent activation of fibroblasts (36). Homeostasis of the ECM is of major importance for the physiological function of most organs, and is maintained by secretion of proteases and degradation of ECM components, as well as synthesis of collagens (37). In addition, both ECM constituents and MMPs may act as modulators of inflammation by regulating barrier function along with cytokine and chemokine activity (25, 38, 39). Even though collagen deposition is an essential step in limiting alveolar damage and initiating repair (40), elevated levels of procollagen (COL3) and proteases (MMP1/3) have also been reported as biomarkers of poor prognosis in human ALI patients (41, 42). Altogether, this indicates that ECM degradation and subsequent collagen synthesis occurred to a greater extent in the absence of PrP^C^ following LPS challenge. Although no significant histopathological differences between genotypes were observed in the acute phase of ALI, abnormal collagen homeostasis may lead to morphological alterations in the later reparative phase.

When analyzed by IPA, we found that genes associated with proinflammatory upstream regulators, such as TNF-α, IL-1β, and IFN-γ, were more activated in PrP^C^-deficient goats. We also observed a lower activation score of the interferon response pathway in the *PRNP*^Ter/Ter^ group, but this reflects that the PrP^C^-deficient control goats are in a primed state with respect to interferon-responsive genes (16), rather than having a reduced activation potential. Interestingly, type I interferons and some of the downstream effector genes are considered as important regulators of lung inflammation (43). Because most PrP^C^ is localized to the cell surface, it has been hypothesized that PrP^C^ binds extracellular proteins and facilitates transmembrane signaling processes (44). For instance, PrP^C^ desensitized cells to TNF-α by restricting the level of available TNF-receptor present at the plasma membrane of neuroectodermal cells (34). We have recently proposed that PrP^C^ protects tissues by modulating certain signaling pathways of innate immunity (17). This is in agreement with murine studies reporting that lack of PrP^C^ exacerbates inflammation in different organ systems (8, 9, 11). Thus, increased expression of MMPs and ECM constituents might demonstrate an aggravated inflammatory damage of the lung parenchyma in goats devoid of PrP^C^.

We observed moderate levels of *PRNP* mRNA in the lungs, in accordance with reports from sheep and cattle (3, 4, 45, 46). After LPS challenge, *PRNP* expression increased slightly (1.6-fold) in *PRNP*^+/+^ goats, indicating a response to inflammatory stimuli. We have earlier published the same tendency in the hippocampus and choroid plexus (17). IHC showed that PrP^C^ was distributed in several cell types throughout the lungs, similar to what has been reported in a bovine study (45). As expected, the absence of PrP^C^ protein was confirmed in *PRNP*^Ter/Ter^ goats.

Systemic LPS challenge was associated with a dramatic change in the lung transcriptome, in which 432 and 596 genes were differentially expressed in normal and PrP^C^-deficient goats, respectively. Besides the genotype-dependent profile in ECM homeostasis and inflammatory regulators, there was a considerable overlap in the molecular response to LPS. Not surprisingly, the majority of the genes were annotated as being involved in the inflammatory and innate immune response, and showed a comparable profile to those reported in other species (47). The most upregulated gene in both genotypes was the acute phase protein *SAA3*, an isoform of the SAA apolipoproteins. Indeed, *SAA3* seems to be a hallmark of acute inflammation in a range of tissues, at least in ruminants (17, 48). Interestingly, *SAA3* may act as a ligand for the TLR4-MD2-complex, and play multiple roles in modulating the innate immune response (49). Four S100 proteins (*S100G, S100A8, S100A9*, and *S100A12)* were also among the most prominently upregulated genes in both genotypes. These low-molecular weight proteins have a Ca^2+^-binding capacity, and as well as being involved in regulation of proliferation, differentiation and apoptosis (50), also have antimicrobial activity against a variety of microorganisms (51). Because *S100A8*/*A9*/*A12* genes are constitutively expressed in neutrophils, as demonstrated by S100A8 IHC in the present study, infiltrating neutrophils probably contribute to the upregulation of these genes in our model (52). Although the exact functions of S100 proteins in lung inflammation remain currently unresolved, at least S100A8/A9 seems to reduce the development of ALI (53). The most downregulated gene, *CYP1A1*, plays a key role in the metabolism of xenobiotics and some endogenous substrates (54). Similarly, systemic LPS suppressed *CYP1A1* expression in the murine liver (55) and lungs (56). The downregulation could result from the formation of reactive oxygen species (ROS) generated by LPS challenge, as ROS have been shown to suppress *CYP1A1* expression *in vitro* (57). Conversely, LPS treatment induced *CYP1A1* expression in cultured human dendritic cells and in the thymus (58), suggesting that tissue-specific differences may occur.

Acute inflammation is a balancing act; although it is an essential component of an appropriate response and recovery from an insult, it may also damage the tissue in which it occurs. The multifocal areas with alveolar hemorrhage, edema and infiltration of inflammatory cells shown histologically, indicate disruption of the alveolar/capillary basement membrane. This is probably due to the massive release of proteases and inflammatory mediators (59, 60). The observed hydrothorax in two PrP^C^-deficient goats most likely represents transudative pleural effusion resulting from compromised integrity of the lung capillaries. Neutrophils were found sequestered within the pulmonary capillaries and some migrated into the alveoli upon LPS challenge. Correspondingly, several genes mapped to neutrophil chemotaxis, such as *S100A8*/*A9*/*A12* proteins and chemokines (*CCL2, CCL5* and *CCL15*), and IPA confirmed the involvement of granulocyte diapedesis. In some areas, the normal septal structure was replaced by fibrinous exudate compatible with hyaline septa or debris from necrotic cells (26). Although the mean lung histopathology score was slightly higher in *PRNP*^Ter/Ter^ goats than in normal goats, the difference was not statistically significant. The magnitude of LPS-effects on inflammation probably mask any putative histopathological differences associated with the demonstrated genotype-dependent differences in gene transcription, and thus would be difficult to detect by semiquantitative evaluation.

The substantial activation of the lung transcriptome and histopathological evidence of lung injury corresponded with the clinical signs observed. The presence of pulmonary intravascular macrophages, specialized in removing circulating bacteria and endotoxins, probably means that small ruminants are particularly sensitive to systemic LPS exposure (61). A significant increase in respiratory rate was observed, peaking at 1 h, and remained elevated the first 7 h postexposure. Moreover, periods of evident respiratory distress were observed in nearly 90% of the animals receiving LPS, indicating temporarily reduced lung function. This is in agreement with a significant enrichment of genes characterized as hypoxia-responsive. Given that only subtle effects have been attributed to PrP^C^, it was not surprising that loss of PrP^C^ did not affect the overall clinical recovery and outcome.

In conclusion, we demonstrated a potent activation of the lung transcriptome that corresponded with morphological and clinical alterations. Although there was a substantial overlap in the response to LPS in both genotypes, increased turnover of ECM and activation of upstream inflammatory regulators were present in PrP^C^-deficient goats. Future studies should investigate whether these alterations affect the later reparative phase of ALI.

## Availability of Data and Material

Original BAM files containing sequencing data are available from the SRA database (SRA accession number SRP116648). Data and material supporting the conclusions are contained within the manuscript and supplementing material.

## Ethics Statement

The animal experiment was performed in compliance with ethical guidelines and approved by the Norwegian Animal Research Authority (ID 5827, 6903, and 7881) with reference to the Norwegian regulation on animal experimentation (FOR-2015-06-18-761).

## Author Contributions

CE, MT, AE, MB, and ØS designed the study and ØS, MR, CE, AE, and MT performed the experiments. ØS carried out laboratory procedures, performed the statistical analysis, and drafted the manuscript. MR performed the PrP^C^ immunohistochemistry. JK performed the ingenuity pathway analysis. All authors have critically read and approved the final manuscript.

## Conflict of Interest Statement

The authors declare that the research was conducted in the absence of any commercial or financial relationships that could be construed as a potential conflict of interest.

## Footnotes

^1^https://www.ncbi.nlm.nih.gov/sites/batchentrez.

## Abbreviations

LPS, lipopolysaccharide; qPCR, quantitative real-time polymerase chain reaction; DEGs, differentially expressed genes; GO, gene ontology; IPA, ingenuity pathway analysis; IHC, immunohistochemistry; IL, interleukin; IFN, interferon; TNF, tumor necrosis factor; TGF, transforming growth factor.
