# Supplementary material for: Goats without Prion Protein Display Enhanced Proinflammatory Pulmonary Signaling and Extracellular Matrix Remodeling upon Systemic Lipopolysaccharide Challenge
Source: Front Immunol. 2017 Dec 6;8:1722. doi: 10.3389/fimmu.2017.01722 (PMC5723645; doi:10.3389/fimmu.2017.01722)
Supplement: Supplementary file 1 [file presentation_1.pdf]

Supplementary Material

Goats without prion protein display enhanced pro-inflammatory pulmonary signaling and extracellular matrix remodeling upon systemic LPS challenge

Salvesen Ø*, Reiten MR, Kamstra JH, Bakkebø MK, Espenes A, Tranulis MA, Ersdal C

*** Correspondence:** oyvind.salvesen@nmbu.no

Table S1. Study groups showing treatment, animal number, mean age, mean weight and gender.

Treatment	Genotype	Animal number	Mean age (months)	Mean weight (kg)	Gender
LPS	*PRNP*$^{+/+}$	8	7,3 (±0,3)	27,3 (±2,7)	8♀
	PRNP$^{Ter/Ter}$	8	6,7 (±1,1)	26,1 (±4,6)	7♀, 1♂
Saline	*PRNP*$^{+/+}$	5	6,7 (±0,2)	21,5 (±3,2)	5♀
	PRNP$^{Ter/Ter}$	5	6,6 (±0,03)	24,9 (±2,8)	5♀
		**26**	**6,9 (± 0,7)**	**25,3 (± 3,9)**	**25♀, 1♂**

Table S2. RNA quality control. Purity was based on $OD_{260/280}$ and $OD_{260/230}$ absorbance reading using DeNovix DS-11 spectrophotometer (Wilmington, USA). RNA integrity was assessed by the Agilent Bioanalyzer system.

Treatment	Group	$OD_{260/280}$	$OD_{260/230}$	Mean RIN	Novogene RIN*
**LPS**	*PRNP*$^{Ter/Ter}$	2.1 (±0.05)	2.2 (±0.08)	8.5 (±0.29)	9.1
	PRNP$^{+/+}$	2.1 (±0.06)	2.2 (±0.03)	8.6 (±0.27)	8.9
**Saline**	*PRNP*$^{Ter/Ter}$	2.1 (±0.11)	2.2 (±0.04)	8.5 (±0.17)	9.0
	PRNP$^{+/+}$	2.1 (±0.07)	2.1 (±0.12)	8.4 (±0.39)	8.8

* Novogene quality control on samples before RNA sequencing. All samples were assessed as being of the best quality level (A level).

Table S3. RNA sequencing - quality control summary

Sample	Raw reads	Clean reads	Clean bases	Error rate (%)	Q20 (%)	Q30 (%)	GC content (%)
Lu_L_Ter	58928706	57240044	8.59G	0.01	98.14	95.58	52.01
Lu_L_Nrm	40942406	39851306	5.98G	0.01	98.16	95.62	51.24
Lu_C_Ter	47429726	46063496	6.91G	0.01	98.10	95.51	51.94
Lu_C_Nrm	41592070	40369834	6.06G	0.01	98.14	95.60	52.12

Table S4. RNA sequencing - overview of mapping status

Sample	Lu_LPS_Ter	Lu_LPS_Nrm	Lu_Ctrl_Ter	Lu_Ctrl_Nrm
**Total reads**	57240044	39851306	46063496	40369834
**Total mapped**	43924351 (76.74%)	31053064 (77.92%)	35377663 (76.8%)	30723965 (76.11%)
**Multiple mapped**	655985 (1.15%)	464171 (1.16%)	482173 (1.05%)	458717 (1.14%)
**Uniquely mapped**	43268366 (75.59%)	30588893 (76.76%)	34895490 (75.76%)	30265248 (74.97%)
**Read-1**	22137382 (38.67%)	15659895 (39.3%)	17887107 (38.83%)	15487734 (38.36%)
**Read-2**	21130984 (36.92%)	14928998 (37.46%)	17008383 (36.92%)	14777514 (36.61%)
**Reads map to '+'**	21626721 (37.78%)	15291053 (38.37%)	17439028 (37.86%)	15125398 (37.47%)
**Reads map to '-'**	21641645 (37.81%)	15297840 (38.39%)	17456462 (37.90%)	15139850 (37.50%)
**Non-splice reads**	26470495 (46.24%)	19304450 (48.44%)	22101289 (47.98%)	19223946 (47.62%)
**Splice reads**	16797871 (29.35%)	11284443 (28.32%)	12794201 (27.78%)	11041302 (27.35%)

Table S5. Primer sequences used for qPCR

Gene ID	Symbol	Gene name	Primer sequences	Ref.
102179831	ACTB	Actin beta	F: 5'TGCCCTGAGGCTCTCTTCCA<br>R: 5'TGCGGATGTCGACGTCACA	[1]
102169975	PRNP	Prion protein	F: 5'GTGGCTACATGCTGGGAAGT<br>R: 5'AGCCTGGGATTCTCTCTGGT	
102185230	IFI6	Interferon alpha inducible protein 6	F: 5'TATCGCTGTTCCTGTGCTACC<br>R: 5'AAGCTCGAGTCGCTGTTTTC	
100860873	CXCL10	C-X-C motif chemokine ligand 10	F: 5'ACGCTGTACCTGCATCGAG<br>R: 5'GCAGGATTGACTTGCAGGA	
102168428	SAA3.2	Serum amyloid A3	F: 5'CTGGGCTGCTAAAGTGATCAGTAAC<br>R: 5'CCCTTGAGCAGAGGGTCTGTGATT	[2]
102182273	S100A9	S100 calcium-binding protein A9	F: GAGATCATGGAGGACCTGGA<br>R: GGCCACCAGCATAATGAACT	
102180100	ITGAM	Integrin Subunit Alpha M	F: CTTGAGGCCTCCACCAAATA<br>R: GCCCAGGTTGTTGAACTGAT	
102177056	CD14	Cluster of differentiation 14	F: CCACCCTCAGTCTCCGTAAC<br>R: GTGTGCTTGGGCAATGTTC	
100860816	IL1B	Interleukin 1 beta	F: GACAACAAGATTCCTGTGGCC<br>R: TCTACTTCCTCCAGATGAAGTGT	
100860872	GAPDH	Glyceraldehyde 3-phosphate dehydrogenase	F: GCAAGTTCCACGGCACAGTC<br>R: CCCACTTGATGTTGGCAGGA	[1]

References

1. Zhang Y, Zhang XD, Liu X, Li YS, Ding JP, Zhang XR, et al. Reference gene screening for analyzing gene expression across goat tissue. Asian-Australasian journal of animal sciences. 2013;26(12):1665-71.
2. Brenaut P, Lefevre L, Rau A, Laloe D, Pisoni G, Moroni P, et al. Contribution of mammary epithelial cells to the immune response during early stages of a bacterial infection to Staphylococcus aureus. Vet Res. 2014;45:16.

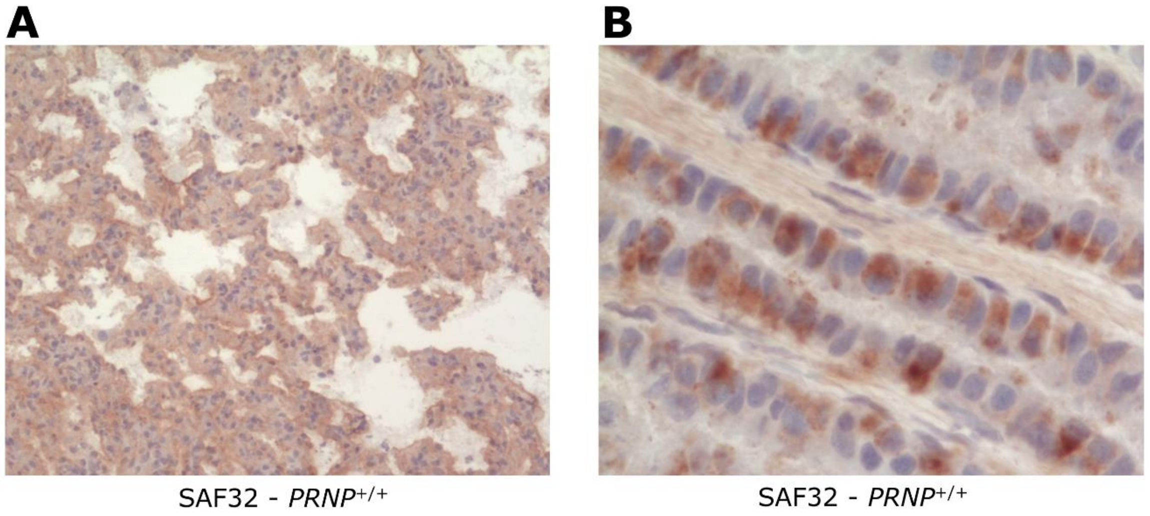

Figure S1. PrP^{C} (SAF32) immunohistochemistry of caprine lung tissue. (A) PrP^{C} labeling is associated with the luminal surface of the alveolar septa. 100 X magnification. **(B)** PrP^{C} shows a granular distribution within bronchial epithelial cells. 400 X magnification. Sections are from representative areas stained with the SAF32 antibody.
